# Co-Morbidities of Irritable Bowel Syndrome in a Racially and Ethnically Diverse Population

**DOI:** 10.3390/jcm13051482

**Published:** 2024-03-04

**Authors:** Christina Lee, Supriya Rao, Howard J. Cabral, Horst Christian Weber

**Affiliations:** 1 Hospital Medicine, Beth Israel Deaconess Medical Center, Boston, MA 02215, USA; clee730@gmail.com; 2 Integrated Gastroenterology Consultants, Lawrence, MA 01841, USA; suprao@gmail.com; 3 Department of Biostatistics, Boston University School of Public Health, Boston, MA 02118, USA; hjcab@bu.edu; 4 Section of Gastroenterology & Hepatology, Boston University Chobanian & Avedisian School of Medicine, Boston, MA 02118, USA

**Keywords:** irritable bowel syndrome, epidemiology, racial and ethnic diversity, co-morbidities, cancer

## Abstract

**Introduction**: Irritable bowel syndrome (IBS) is a disorder of gut–brain interaction (DGBI), and associated co-morbidities worsen quality of life. Research concerning IBS co-morbidities in different racial/ethnic groups is very sparse. This study aimed to determine the prevalence rates of co-morbidities and possible differences in a multiracial/ethnic IBS cohort. **Methods**: Based on ICD-9-coded IBS diagnosis, 740 outpatients (≥18 years) were included in this retrospective study at Boston Medical Center. Demographics and ICD-9-coded co-morbidities were extracted from electronic records. Descriptive statistics and multiple logistic regression were used for data analyses. **Results**: The most prevalent co-morbidities in this IBS cohort included gastroesophageal reflux disorder (GERD) (30%), depression (27%), anxiety (23%), (chronic obstructive pulmonary disease) COPD/asthma (16%), and obesity (10%). GERD was more prevalent in Hispanics and Blacks (*p* = 0.0005), and non-ulcer dyspepsia (NUD) was more prevalent in Blacks and Asians (*p* = 0.003). Higher rates of diabetes mellitus type 2 (DMT2) (*p* = 0.0003) and depression (*p* = 0.03), but not anxiety (*p* = 0.9), were present in Blacks and Hispanics. GERD was significantly associated with Hispanics (*p* = 0.003), dependent on age, overweight, and obesity. NUD was significantly associated with Blacks (*p* = 0.01) and Asians (*p* = 0.006), independent of sex, age, and BMI. Cancer of the thyroid, ovaries, and testis occurred at a five-fold higher rate than expected. **Conclusions**: Significant racial/ethnic differences exist for IBS co-morbidities in this study cohort, including depression, DMT2, GERD, and NUD. Certain cancers were found to be more frequent in this IBS sample as compared with the general population.

## 1. Introduction

Irritable bowel syndrome (IBS) is one of the most common disorders of the gut–brain (DGBI) interactions, formerly referred to as functional gastrointestinal disorders (FGIDs), characterized by abdominal pain with altered bowel habits in the absence of structural and biochemical abnormalities [1,2]. IBS is estimated to affect around 1 in 10 people globally and affects up to 12% of the North American population [3]. The Rome criteria, a symptom-based criteria established in the late 1980s, continues to be used for the diagnosis of IBS [4]. Per the most recent Rome IV criteria, IBS subtypes are classified by the predominant bowel pattern and include the following: IBS with diarrhea (IBS-D), IBS with constipation (IBS-C), IBS with mixed bowel habits (IBS-M), and un-subtyped IBS (IBS-U) [5,6].

It has long been known that IBS has a substantially negative effect on the quality of life (QoL) of patients [7,8], with non-white IBS patients reporting significantly worse health-related QoL (HRQOL) compared with the general US population [9]. Likewise, in a multinational study, FGIDs were associated with more frequent doctor visits and lower QoL [10]. Specifically, IBS patients have the same physical HRQOL as diabetic patients and a lower physical HRQOL compared with patients who have depression or GERD [11]. It has also been suggested that longer-term QoL may be affected more by psychological well-being than by the improvement in gastrointestinal symptom severity [12,13], with this understanding highlighting the importance of a holistic approach to IBS care [2]. In another cross-sectional health survey study based in the USA, Canada, and the UK, FGID presence in multiple regions (esophageal, gastroduodenal, bowel, and anorectal) correlated with more medical therapies and a higher prevalence of abdominal surgeries, leading to worse mental/physical QoL (*p* < 0.001) [14]. As an extension to this prior study, a global survey also demonstrated decreased QoL with an increasing number of DGBI regions (esophageal, gastroduodenal, bowel, and anorectal) affected (*p* < 0.0001) [15].

Due to the continued large illness burden of IBS on the individual patient, there is also a corresponding larger healthcare system burden with healthcare costs in the USA [16]. Compared with non-IBS patients, IBS patients had higher inpatient admissions, outpatient visits, and outpatient prescriptions, resulting in higher total costs and healthcare system burden [17].

DGBIs, specifically IBS, are associated with a series of non-gastrointestinal and gastrointestinal co-morbidities and other functional gastrointestinal disorders [18]. Co-morbid or extraintestinal symptoms occur frequently with IBS and account for up to three-fourths of excess healthcare visits [18]. Specifically, psychological co-morbidities, including depression and anxiety, are frequently associated with IBS, occurring in up to 94% of IBS patients [2,18].

There is also a myriad of non-gastrointestinal, non-psychiatric disorders associated frequently with IBS, according to Whitehead et al., including fibromyalgia, chronic fatigue syndrome, temporomandibular joint disorder, and chronic pelvic pain. Other co-morbidities with high prevalence also include headache, chronic neck and back pain, hypertension, and diabetes mellitus, all of which demonstrated association with IBS even after adjusting for demographic characteristics and D5M-IV mental disorders [19].

There are limited research data suggesting that IBS does not increase the overall risk of cancers and that IBS lowers the risk of incident colorectal cancer and colorectal polyps [20,21]. In contrast, conflicting results suggest an increased risk of colorectal cancer in IBS patients, primarily in the first year of IBS diagnosis [22]. Wu et al. suggested that an increased risk of colorectal cancer within one year of IBS diagnosis may be due to misclassifications resulting from overlapping symptoms rather than causation [23]. Beyond the colon cancer studies, there are no other reported malignancy associations with IBS [19].

While there is a substantial body of literature regarding IBS-associated gastrointestinal, psychiatric, and somatic co-morbidities, research in multiracial/ethnic populations with IBS is very limited despite racial/ethnical differences being recognized in few studies on IBS epidemiology, symptom presentation, quality of life, and healthcare utilization [9,24,25,26]. Furthermore, studies on cancer rates in general IBS populations are very sparse and demonstrate a significant knowledge gap [27]. Research of IBS-associated co-morbidities, including cancers across racial/ethnic groups, has not been conducted thus far to our knowledge in dedicated studies.

Therefore, the aim of our study was to determine the estimated prevalence rates and demographic characteristics of selected co-morbidities, including cancer, in IBS patients in a racially diverse large safety net outpatient hospital that delivers healthcare services to vulnerable patient populations. The results from our study should provide important data to better serve a multiracial IBS population with more directed management of DGBI to improve outcomes, such as QoL and healthcare utilization.

## 2. Materials and Methods

### 2.1. Patient Population

We performed a retrospective, cross-sectional study at the Boston Medical Center (BMC), the academic medical center of Boston University Chobanian & Avedisian School of Medicine, serving as the largest tertiary safety net hospital in New England.

This study was approved by the BMC Institutional Review Board (IRB) and was in accordance with the ethical standards of the institutional and national research committee and with the 1995 Helsinki Declaration and its later amendments or comparable ethical standards. Due to the nature of this retrospective, population-based study and the preserved anonymity of patients, a waiver of informed consent was obtained from and approved by the BMC IRB.

A total of 740 patients (ages ≥ 18 years) with the ICD-9 diagnosis of IBS (564.1) between 2005 and 30 September, 2007 were included in this study from a review of the electronic medical records. IBS co-morbidities were identified based on ICD-9 coding.

### 2.2. Outcome Measures

The primary outcome of this study was to determine the prevalence of various IBS-related co-morbidities across racial/ethnic groups represented and IBS subtypes, including IBS-Constipation (IBS-C), IBS-Diarrhea (IBS-D), IBS-Mixed (IBS-M), and IBS-Unsubtyped (IBS-U) according to the Rome III criteria [28]. The IBS subtype for each identified study patient was determined based on chart review by three study investigators using Rome III criteria [28]. The interrater reliability was determined using kappa statistics in a test sample (kappa > 0.8). The putative IBS-related co-morbidities included in this study were selected from other frequent DGBI, such as non-ulcer dyspepsia (NUD), GI disorders (GERDs), psychiatric disorders (anxiety and depression), and generally highly prevalent conditions (chronic obstructive pulmonary disease (COPD)/asthma, obesity, diabetes mellitus type 2 (DMT2)), and cancer recorded in patients’ records.

The secondary outcome of this study was to identify statistically significant racial/ethnic and demographic differences in this IBS patient sample with certain co-morbidities.

### 2.3. Statistical Analysis

In order to examine the univariate distributions of the data, we generated descriptive statistics using counts and percentages for categorical variables and means, standard deviations, and minima and maxima for continuous variables. We next performed bivariate analyses using chi-square and Fisher’s exact tests of significance for crosstabulations of categorical variables, e.g., sex, marital status by IBS subgroup or cancer status, and two sample *t*-tests and one-way analyses-of-variance (ANOVA) with post hoc comparisons following statistically significant global tests via Tukey’s studentized range procedure for continuous dependent variables by categorical variables, e.g., mean age, BMI by cancer status, and IBS subgroup. We also compared the observed rates for individual cancer diagnoses in the IBS sample along with their 95% exact confidence intervals to published data from SEER on the age-adjusted prevalence of the diagnoses. Those intervals that included the SEER rates were deemed to be consistent with the general population prevalence of the diagnosis corresponding to the duration of the retrospective study. Logistic regression analyses were performed for selected patients’ characteristics and GI co-morbidities. All of the analyses were performed using SAS for Windows, version 9.4 (ref) 2008 SAS Institute, Inc., Cary, NC, USA. *p*-values less than 0.05 were deemed statistically significant.

## 3. Results

### 3.1. IBS Study Population and Co-Morbidities

A total of 740 IBS patients were enrolled in the study based on the ICD-9 diagnosis (564.1) in the electronic medical record system at the BMC. Forty patients did not indicate their race/ethnicity and thus were not considered for further analyses when race/ethnicity was considered. Of those patients, 492 were White (70%), 99 were Black (14%), 44 were Asian (6%), and 65 were Hispanic (9%). The basic demographic characteristics of the entire IBS sample are shown in the left column of Table 1. The median age of all IBS patients was 40 years old with the majority (67%) of the cohort being younger than 50 years of age. The diagnosis of IBS was established on average 7 years earlier, and there was a strong female predominance (75%). The majority of IBS patients (69%) had private insurance. Limited data on the educational level showed that 91% had a college and/or graduate degree.

The most common co-morbidities overall in IBS patients included gastroesophageal reflux disease (GERD), with 30%; depression, with 27%; anxiety, with 23%; (chronic obstructive pulmonary disease) COPD/asthma, with 16%; and obesity, with 10%. As seen in Table 2, GERD, depression, and diabetes mellitus type 2 (DMT2) were all significantly more prevalent in Hispanic and Black patients compared with Asian and White patients in this IBS cohort. NUD was significantly more prevalent in Black and Asian patients.

### 3.2. GERD and NUD

The details of IBS patients with and without GERD and NUD are summarized in Table 3, Table 4, Table 5 and Table 6. GERD and NUD occurred in 30% and 7% of all IBS patients, respectively. The frequencies of IBS subtypes were similar in IBS patients with and without GERD and NUD (Table 3 and Table 4). However, IBS patients with and without GERD and NUD demonstrated significant racial/ethnic differences. Additional analyses via multiple logistic regression models revealed that GERD was significantly associated with Hispanics when compared with White patients, independent of sex but dependent on age and body mass index (BMI), with GERD associated with age >30 and overweight/obese patients (Table 5). In contrast, NUD was significantly associated with Blacks and Asians when compared with White patients, independent of sex, age, and BMI (Table 6).

### 3.3. Cancer in IBS Patients

The demographic data of IBS subjects with and without cancer are summarized in Table 1. There were 28 patients (4%) in the entire IBS sample (*n* = 740) who were diagnosed with a total of 31 cancers, including 24 solid tumors, six skin cancers, and one lymphoma. Patients with IBS and concurrent cancer were diagnosed with IBS at a significantly older median age of 60 years compared to patients with IBS and no concurrent cancer (40 years; *p* < 0.0001). Patients with IBS and cancer had higher prevalence of Medicaid/Medicare insurance rates (39% vs. 16%, *p* = 0.008). The racial breakdown (Table 1; *p* = 0.6) and IBS subtype distribution was not different between IBS with and without cancer (Table S1).

As demonstrated in Table 7, estimated prevalence rates of thyroid, ovary cancer, testis, and Hodgkin’s lymphoma in this IBS sample were substantially increased when compared with the historic, age-adjusted SEER data corresponding to the time frame during which the IBS data collection was conducted; however, other cancers, such as colon carcinoma, were not different. Most cancers were diagnosed prior to IBS, with a median elapsed time of 11.5 years (range 1–39) (Table S2).

## 4. Discussion

The major strength of this large retrospective study conducted in a tertiary care safety net hospital is the unique racial/ethnic diversity of the IBS patient cohort representing different low-income and minority populations. It is one of the very few studies of selected co-morbidities, including concurrent cancer, in a racially/ethnically mixed IBS outpatient population that analyzes and addresses significant knowledge gaps. The results of this study are likely to enhance our ability to improve outcomes in IBS patients of varied racial/ethnic backgrounds.

Patients with IBS, now referred to as DGBI, commonly present with various symptoms and co-morbidities beyond the gastrointestinal (GI) symptoms, which account for a substantial portion of the clinical and economic burden [29]. The high level of symptom burden leads to twice as many healthcare visits for IBS patients compared with age-matched controls, and 78% of extra healthcare visits from IBS patients are due to the extraintestinal manifestations/co-morbidities rather than the GI symptoms [30,31].

In addition to the societal burden of the healthcare system, IBS continues to have a substantial negative impact on the QoL of individual IBS patients. With an increased number of co-morbidities, the QoL and psychological distress worsen for patients; with an increased burden of extraintestinal and psychiatric co-morbidities, the GI symptoms in IBS are more severe and more resistant to treatment, leading to a vicious cycle of seemingly untreated IBS GI symptoms, IBS-related co-morbidities, and reduced QoL [18,32,33,34,35,36,37]. Classification systems of IBS and treatment guidelines do not take into account extraintestinal co-morbidities and/or psychological symptoms [6,38]. Therefore, it is imperative for physicians to also consider the IBS-related co-morbidities for a more holistic, individualized, and effective treatment plan.

IBS is well known to be specifically associated with high prevalence rates of psychological disorders [2]. As demonstrated in a meta-analysis, IBS patients had significantly higher anxiety and depression levels than controls (*p* < 0.01) [39]. Another study showed that both anxiety and depression were predictors of the development of IBS, and IBS patients without anxiety and depression at baseline reported higher levels of these conditions at follow-up [40].

In this large study of 740 IBS outpatients at a large safety net hospital, we analyzed selected co-morbidities in a racially/ethnically diverse sample (Table 1), which demonstrated significant differences for gastrointestinal, non-gastrointestinal, and psychiatric co-morbidities across racial/ethnic groups. GERD was found to be significantly more frequent in Hispanic and Black patients, whereas NUD was more frequent in Asian and Black IBS patients (*p* = 0.0005 and *p* = 0.004; Table 3 and Table 4, respectively). Significant racial/ethnic differences were also evident with higher rates of DMT2 (*p* = 0.0003) and depression (*p* = 0.03), but not anxiety (*p* = 0.9), in Blacks and Hispanics (Table 2). While it was not the goal of this study to examine possible socio-economic disadvantages as underlying factors for these differences, as previously suggested, this avenue of investigation deserves more attention [41,42]. Unlike our current study with an overall rate of 5% DMT2, a recent survey of IBS patients reported a higher prevalence of DMT2 at 16% [19]; however, our findings demonstrate a significantly higher rate of DMT2 in the Hispanic and Black patients at 8% and 14%, respectively (*p* = 0.0003). In contrast to these previously unrecognized race/ethnicity-based differences of IBS co-morbidities, no differences were identified based on the presenting IBS subtype (Table S3). In view of the possible negative impact of co-morbidities on IBS diagnosis and severity, our data underscore the importance of clinical screening and aggressive treatment of such conditions, specifically in low-income and minority populations.

GERD occurred overall in 30% of all study patients, which correlates with the reported range of the well-known GERD-IBS overlap between 10 and 74% based on endoscopic diagnosis [43,44]. Different mechanisms, such as visceral hypersensitivity or dysmotility with delayed gastric emptying in affected patients, smooth muscle dysfunction of the GI tract, or impaired duodenal acid clearance, have been considered [45,46,47,48,49,50].

In our study, GERD was significantly more prevalent in Hispanic IBS patients at 51% compared with other racial/ethnic groups (*p* = 0.0005), but GERD was not associated with IBS subtypes (Table 3). Additional analyses by multiple logistic regression models confirmed that GERD was significantly associated with Hispanics, with age above 30 and overweight/obesity, when compared to White patients (Table 5, *p* = 0.003). A recent study found that Hispanics were 2.44 times more likely to have persistent symptoms while on proton pump inhibitor therapy when compared to non-Hispanic white patients, and IBS patients were more likely to have PPI-refractory GERD [51]. The combined significance of GERD in Hispanic IBS patients remains elusive.

Furthermore, NUD, or functional dyspepsia, occurred in 7% of all IBS patients in our study, with a significantly higher prevalence in Black and Asian patients at 13% and 14%, respectively (*p* = 0.003), but NUD was not associated with IBS subtypes (Table 4). Multiple logistic regression confirmed that NUD was associated more frequently with Blacks and Asians when compared with White patients (Table 6; *p* = 0.01 and *p* = 0.006, respectively), independent of sex, age, and BMI. These findings represent novel insights into GI disorders and DGBI in a racially/ethnically diverse IBS population, which have not been previously reported.

Research regarding concurrent cancer in IBS patients is extremely sparse; one recent national survey did not find increased cancer rates associated with IBS (OR 0.53, 95% CI: 0.23–1.18) [19]. In a Danish study, a decreased risk of colorectal cancer was reported in the 1–10-year period after an IBS diagnosis, but in the first 3 months after an IBS diagnosis, the risk of colon cancer was more than eight-fold increased, and the risk of rectal cancer was five-fold increased [52]. We detected co-morbidity cancer at an overall 4% prevalence rate in our IBS cohort (Table 1 and Table 2), with a total of 31 cancers in 28 patients without differences across the racial/ethnic groups and not affected by IBS subtype (Table S3). Our study findings represent a two-fold higher rate than the reported 1.78% by the CDC for the years between 2015 and 2020 for all cancers in all age groups [53]. Not surprisingly, IBS patients with concurrent cancer were significantly older (60 years median age) than patients with IBS without cancer (median age 40 years; Table 1; *p* < 0.0001) in our study.

Patients with IBS and cancer (Table 1) had a higher prevalence of Medicaid/Medicare insurance (39% vs. 16%, *p* = 0.008), which might reflect lower socio-economic status of these patients, leading to reduced/untimely access to healthcare. Notably, our study data were collected between 2005 and 2007. Universal healthcare coverage in Massachusetts was established between this time period in 2006–2007 and was not included in our IBS cohort. For future analysis, it would be of great interest to analyze the impact of statewide insurance expansion on the IBS patient population. Comparison of the educational level, however, did not differ between these two groups but was limited by a large number of missing data (Table 1; *p* = 0.15).

When compared with the corresponding SEER database (1975 to 2005) age-adjusted specific cancer rates during the study period, cancer rates in the IBS patient sample in this study, including thyroid, ovarian, and testicular cancer, demonstrated a more than five-fold increase (Table 7). To date, there is no direct association between IBS and specific cancers reported in the literature, and our findings may be explained by the retrospective design of this study, a relatively small sample size, rare cancer events, referral bias at the study site for certain disorders, and other confounding factors yet unidentified. Nevertheless, larger prospective studies are warranted to investigate the hypothesis of IBS-associated cancers.

There are several significant limitations of this study. First, in this retrospective chart review study, patients were enrolled based on an ICD-9 IBS code only, and the diagnoses could not be confirmed using appropriate, validated IBS questionnaires such that non-IBS patients may have been misclassified in this study. Secondly, the IBS subtype assignment was also not based on validated questionnaires but only on careful chart review—although with excellent interobserver correlation. Thirdly, the co-morbidities investigated were selected and not comprehensively or systematically included to represent GI, non-GI, psychiatric disorders, and cancers. Lastly, demographic characteristics such as alcohol use and diet were not included in the study but can be included in future analyses.

## 5. Conclusions

In aggregate, we present the first large-scale study regarding co-morbidities in a racially/ethnically diverse outpatient IBS population with several novel findings. We document specific patterns of racial healthcare differences in specific, prevalent GI, non-GI, and psychiatric disorders negatively affecting Black and Hispanic IBS patients most often. Since co-morbidities in IBS most often lead to negative outcomes in symptom severity, QoL, and healthcare utilization, the findings of this study should enable clinicians to better identify and treat conditions with a more holistic IBS management approach.

## Figures and Tables

**Table 1:** Demographic characteristics of IBS patients with and without cancer at the BMC.

Characteristics	All IBS (*n* = 740)	IBS with Cancer (*n* = 28)	IBS without Cancer (*n* = 712)	*p* Value ^†^
Median age [range] Median BMI [range] Median age at IBS dx [range]) Current age (%): <30 30–49 50+ Gender (%): Male Female Race/ethnicity (%): * White Black Hispanic Asian Missing (*n* = 40) Marital status (%): * Married Single Divorced Widowed Missing (*n* = 20) With children (%): * Yes No Missing (*n* = 175) Smoking Hx (%): * Current Former Never Missing (*n* = 136) Insurance type (%): * Private insurance Medicaid/Medicare None/Free Care Missing (*n* = 1) Highest level of education (%): * Pre high school High school College Graduate/Professional Missing (*n* = 535) Employment (%): * Unemployed Full time Part time Unspecified **	40 (19–89] 25.4 (14.3–64.2] 33 (10–89] 27 40 33 25 75 70 14 10 6 — 32 56 11 1 — 38 62 — 18 16 66 — 69 16 15 — 2 7 41 50 — 7 29 3 61	60 (29–83] 25.6 (16.8–47.3] 57 (36–75] 3 18 79 32 68 74 19 7 0 — 41 22 37 0 — 57 43 — 18 36 46 — 54 39 7 — 0 33 0 67 — 7 39 0 54	40 (19–89] 25.4 (14.3–64.2] 32 (10–89] 27 41 32 25 75 70 14 9 7 — 32 57 10 1 — 32 68 — 17 16 67 — 69 16 15 — 1 7 42 50 — 7 29 3 61	<0.0001 ^†^ 0.57 ^†^ <0.0001 ^†^ <0.0001 ^†††^ 0.37 ^††^ 0.60 ^†††^ 0.0001 ^†††^ 0.006 ^††^ 0.07 ^†††^ 0.008 ^††^ 0.15 ^†††^ 0.60 ^†††^

*n* = 740 is the total of IBS patients (age ≥18 yrs) in the cohort. *n* = 28 is the total of IBS patients with cancer, which is 4% of the entire IBS sample (*n* = 740) who were diagnosed with a total of 31 cancers, including 24 solid tumors, 6 skin cancers, and 1 lymphoma. *n* = 712 is the total number of IBS patients without cancer. The reported numbers shown are percentages, which add up to a total of 100% in each column. For the BMI data, 527 were available from all 740 patients. Of the IBS with cancer patients (*n* = 28), 26 have BMI data, and 501 of all IBS non-cancer patients (*n* = 712) have BMI data. For the age at diagnosis, data of 437 of all 740 patients were available, with 15 (of 28) for IBS with cancer and 422 for IBS without cancer patients (of 712). * Missing data refers to the total IBS cohort of 740 patients only. ** Unspecified employment also includes students. ^†^ By 2-sided Wilcoxon rank sum test. ^††^ By chi-square test; ^†††^ By Fisher’s exact test.

**Table 2:** IBS co-morbidities by race (%) *.

Co-Morbidities	All IBS (*n* = 740)	White (*n* = 492)	Black (*n* = 99)	Hispanic (*n* = 65)	Asian (*n* = 44)	*p* Value ^†^
GERD ** NUD ** Depression Anxiety Obesity COPD/asthma ** DMT2 ** Cancer	30 7 27 23 10 16 5 4	27 5 26 23 9 15 3 4	33 13 33 22 16 18 14 5	51 9 32 25 11 20 8 3	23 14 11 18 5 14 2 0	0.0005 0.003 ^††^ 0.03 0.9 0.1 0.7 0.0003 ^††^ 0.6

* IBS sample comparison by race: missing race data *n* = 39; 1 Native American was not included. The reported numbers are represented in percentages (%). ** GERD = gastroesophageal reflux disease; NUD = non-ulcer dyspepsia; COPD = chronic obstructive pulmonary disease; D MT2 = diabetes mellitus type 2. † By chi-square test. †† By Fisher’s exact test.

**Table 3:** GERD vs. non-GERD in IBS sample by IBS subtype and race/ethnicity (%).

	IBS		*p* Value ^†^
	GERD Present	GERD Absent	
IBS subtype: * IBS-D IBS-C IBS-M IBS-U	29 32 33 29	71 68 67 71	0.80
Race/ethnicity: ** White Black Hispanic Asian	27 33 51 23	73 67 49 77	0.0005

* Data refer to all IBS: *n* = 740. IBS with GERD: *n* = 224. IBS without GERD: *n* = 516. The reported numbers shown are percentages, which add up to a total of 100% in each row. ** Data refer to *n* = 700; missing data for race in IBS sample 39; 1 Native American was not included in the IBS sample. IBS with GERD: *n* = 207. IBS without GERD: *n* = 493. † Chi-square test.

**Table 4:** NUD vs. non-NUD in IBS sample by IBS subtype and race/ethnicity (%).

	IBS		*p* Value ^†^
	NUD Present	NUD Absent	
IBS subtype: * IBS–D IBS–C IBS–M IBS–U	8 8 8 5	92 92 92 95	0.55
Race/ethnicity: ** White Black Hispanic Asian	5 13 9 14	95 87 91 86	0.004

The reported numbers shown are percentages, which add up to a total of 100% in each row. * Data refer to all IBS: *n* = 740. IBS with NUD: *n* = 52. IBS without NUD: *n* = 688. ** Data refer to *n* = 700; missing data for race in IBS sample *n* = 39; 1 Native American was not included into IBS sample. IBS with NUD: *n* = 48. IBS without NUD: *n* = 652. † Chi-square test.

**Table 5:** GERD vs. non-GERD in IBS sample by multiple logistic regression analysis †.

	Odds Ratio (95% CI *)	*p* Value
Gender: female vs. male Race/Ethnicity: Black vs. White Hispanic vs. White Asian vs. White Age group (in yrs): 30–49 vs. <30 50+ vs. <30 BMI: Underweight vs. normal Overweight vs. normal Obese vs. normal	1.58 (0.98, 2.54) 1.02 (0.60, 1.72) 2.62 (1.39, 4.92) 1.33 (0.56, 3.16) 2.32 (1.31, 4.09) 2.59 (1.46, 4.59) 0.56 (0.18, 1.78) 1.88 (1.15, 3.07) 1.74 (1.05, 2.89)	0.06 0.95 0.003 0.51 0.004 0.001 0.33 0.01 0.03

† Data refer to IBS patients with complete information on BMI (*n* = 501); IBS with GERD *n* = 166; IBS without GERD *n* = 335. Model *p* < 0.0001, c statistic = 0.67, Hosmer–Lemeshow *p* = 0.79. BMI < 19.0 is underweight, 19 to 24.9 is normal, 25.0 to 29.9 is overweight, and ≥30.0 is obese. * CI: confidence interval.

**Table 6:** NUD vs. non-NUD in IBS sample by multiple logistic regression analysis †.

	Odds Ratio (95% CI *)	*p* Value
Gender: female vs. male Race/Ethnicity: Black vs. White Hispanic vs. White Asian vs. White Age group (in yrs): 30–49 vs. <30 50+ vs. <30 BMI: Underweight vs. normal Overweight vs. normal Obese vs. normal	1.72 (0.68, 4.40) 3.04 (1.31, 7.04) 2.71 (0.91, 8.08) 4.95 (1.58, 15.50) 1.01 (0.39, 2.60) 1.19 (0.46, 3.07) 0.45 (0.06, 3.60) 0.57 (0.22, 1.48) 0.84 (0.35, 2.02)	0.25 0.01 0.07 0.006 0.99 0.72 0.45 0.25 0.70

† Data refer to IBS patients with complete information on BMI (*n* = 501); IBS with NUD *n* = 36; IBS without NUD *n* = 465. Model *p* = 0.08, c statistic = 0.68, Hosmer–Lemeshow *p* = 0.41. BMI < 19.0 is underweight, 19 to 24.9 is normal, 25.0 to 29.9 is overweight, and ≥30.0 is obese. * CI: confidence interval.

**Table 7:** Cancer prevalence rates in IBS patients at the Boston Medical Center †.

Type of Cancer	Observed Number of Patients with Cancer (Age Range in yrs at Cancer dx)	Expected Number of Patients with Cancer	Cancer Rate in IBS (95% Exact Confidence Interval)	SEER Age-Adjusted Prevalence (All Ages) % **	Fold Increase of Cancer Rates in IBS Compared to SEER Age Adjusted Prevalence
Breast (of 555 women) Prostate (of 185 men) * Thyroid Malignant melanoma * Basal cell ca (skin) * Colon Lung Ovary (of 555 women) Testis (of 185 men) Hodgkin’s lymphoma Urinary bladder Ureter Cervix (of 555 women) *	6 (53–73) 4 (54–63) 4 (21–49) 3 (27) 3 (44) 2 (56–64) 2 (35–48) 2 (30–31) 1 (63) 1 (28) 1 (57) 1 (57) 1 (-)	6 3 0.6 1 not available 2 0.7 0.3 0.1 0.2 1 not available 0.5	1.08 (0.40, 2.34) 2.16 (0.59, 5.44) 0.54 (0.15, 1.38) 0.41 (0.08, 1.18) 0.41 (0.08, 1.18) 0.27 (0.03, 0.97) 0.27 (0.03, 0.97) 0.36 (0.04, 1.30) 0.54 (0.01, 2.97) 0.14 (<0.01, 0.75) 0.14 (<0.01, 0.75) 0.14 (<0.01, 0.75) 0.18 (<0.01, 1.00)	1.0717 1.5073 0.0783 0.1453 not available 0.2696 0.0967 0.0648 0.0669 0.0278 0.1228 not available 0.0817	1.0 1.4 6.9 2.8 not available 1.0 2.8 5.6 8.1 5.0 1.1 not available 2.2

† 28 patients with IBS were diagnosed with 31 cancers. Three of these patients were diagnosed with two simultaneous cancers: one patient—Hodgkin’s lymphoma and breast cancer; one patient—ureteral and urinary bladder cancers; one patient—colon and prostate cancers. * Missing data about patient age at cancer dx: prostate ca—1 patient, malignant melanoma—2 patients, basal cell carcinoma (skin)—2 patients, cervix ca—1 patient. ** SEER Cancer Statistics Review, 1975–2005. http://seer.cancer.gov/csr/1975_2005/sections.html (accessed on 13 December 2023).

## Data Availability

The data is contained within the article and Supplementary Materials.

## Supplementary Materials

The following supporting information can be downloaded at: https://www.mdpi.com/article/10.3390/jcm13051482/s1, Table S1: IBS Subtypes in IBS patients with and without cancer; Table S2: Temporal relation between IBS and cancer diagnosis; Table S3: IBS co-morbidities by IBS subtype.
