# Supplementary material for: Co-Morbidities of Irritable Bowel Syndrome in a Racially and Ethnically Diverse Population
Source: J Clin Med. 2024 Mar 4;13(5):1482. doi: 10.3390/jcm13051482 (PMC10934174; doi:10.3390/jcm13051482)
Supplement: Supplementary file 1 [file jcm-13-01482-s001.zip › jcm-2800619-supplementary.pdf]

Supplemental Digital Contents

Table S1: IBS Subtypes in IBS Patients with and without Cancer (%)

Subtype*	All IBS (N=740)	IBS with cancer (N=28)	IBS without cancer (N=712)	P value†
				0.22
IBS-D	25	25	25	
IBS-C	22	11	22	
IBS-M	13	7	13	
IBS-U	40	57	40	

* IBS–D= IBS with diarrhea, IBS–C= IBS with constipation, IBS–M= IBS with mixed bowel habits, IBS–U= unsubtyped IBS. IBS subtype was determined as described in the "Methods" section.

† Chi-square test.

This table shows that no differences (p=0.22) are detected of IBS subtype frequencies in patients with and without cancers.

Table S2: Temporal Relation between IBS and Cancer Diagnosis†		
	Type of cancer	Cancer dx in years prior or post IBS dx.
Prior IBS*	1. Breast cancer	8
	2. Breast cancer	5
	3. Breast cancer	4
	4. Lung cancer	1
	5. Prostate cancer	12
	6. Ovary cancer	39
	7. Ovary cancer	16
	8. Colon cancer	2
	9. Thyroid cancer	17
Post IBS**	1. Prostate cancer	2
	2. Basal cell carcinoma (skin)	1

† Data of IBS or cancer diagnosis time missing: N=16
* Median of time elapsed from cancer dx to IBS dx: 11.5 years, range 1-39.
** Median of time elapsed from IBS dx to cancer dx: 1.5 years, range 1-2.

This table summarizes the temporal relationship between the IBS and cancer diagnosis. Most cancers were detected prior to the IBS diagnosis.

Table S3: IBS Co-morbidities by IBS subtype (%)

Co-morbidities	All IBS (N=740)	IBS-D (N=187)	IBS-C (N=159)	IBS-M (N=96)	IBS-U (N=298)	P value †
GERD*	30	29	32	33	29	0.8
NUD*	7	8	8	8	5	0.6
Depression	27	25	25	31	26	0.7
Anxiety	23	24	21	21	23	0.9
Obesity	10	12	6	9	10	0.2
COPD/asthma*	16	18	13	10	17	0.2
DM type 2*	5	5	5	6	5	0.96
Cancer	4	4	2	2	5	0.2

*GERD = gastroesophageal reflux disease; NUD = nonulcer dyspepsia; COPD = chronic obstructive pulmonary disease; DM type 2 = diabetes mellitus type 2.
† Chi-square test.

This table shows prevalence rates (%) of co-morbidities in IBS patients according to IBS subtype assignment. There are no differences of co-morbidity rates noted between IBS subtypes.
